# Mechanism of action of cisplatin on Na^+^/K^+^ ATPase of Caco-2 colon cells in vitro

**DOI:** 10.1371/journal.pone.0342707

**Published:** 2026-02-24

**Authors:** Rida Mourad, Rawad Hodeify, Sawsan Kreydiyyeh

**Affiliations:** 1 Department of Biology, Faculty of Arts & Sciences, American University of Beirut, Beirut, Lebanon; 2 Department of Biotechnology, School of Arts & Sciences, American University of Ras Al Khaimah, Ras Al Khaimah, United Arab Emirates; Zhejiang Cancer Hospital, CHINA

## Abstract

Cisplatin is a chemotherapeutic agent that reverts cancerous cells to the apoptotic route. A decrease in the activity of the Na^+^/K^+^ ATPase is one of the hallmarks of apoptosis and a major causative factor of the drug-induced nephrotoxicity. Whether cisplatin targets also the colonic ATPase is a question that was addressed in this work using Caco-2 cells as a model. ATPase activity was measured via inorganic phosphate release with and without ouabain, and protein expression was assessed by Western blot. Cisplatin reduced the activity of the Na^+^/K^+^ ATPase, an effect that was dependent on the transmembrane chloride gradient but had no effect on the purified enzyme, suggesting an indirect action. Fluorescence imaging showed a decrease in the membrane ATPase abundance. Cisplatin was shown to act by increasing intracellular calcium, triggering a sequential activation of p38MAPK and PKA that results in JNK inhibition and a decrease in the Na^+^/K^+^ ATPase activity.

## Introduction

Cancer remains a pressing global health concern, affecting millions worldwide. Among the common chemotherapeutic agents, cisplatin—also known as cis-diaminedichloroplatinum (CDDP)—is one of the most widely used. This platinum-based coordination compound features two chlorides and two ammonias as ligands and has proven effective in combating various cancers. The mechanism of cisplatin’s transport across cell membranes remains however contentious. Research supports both passive and active transport processes, with evidence pointing to the involvement of copper transporters as well as endocytosis and transport via organic cation transporters [1]. Once inside the cell, cisplatin undergoes aquation. Its chloride ligands are replaced by water molecules generating reactive platinum species that bind DNA, inflicting damage on cancerous cells and ultimately triggering apoptosis [2]. Cisplatin can also induce apoptosis and tissue necrosis by disrupting calcium homeostasis, which leads to mitochondrial dysfunction and depletion of adenosine triphosphate (ATP) [3,4].

Despite its efficacy, cisplatin use is marred by challenges, particularly the emergence of resistance. Mechanisms of resistance include altered drug transport across cell membranes, defects in apoptotic signaling pathways, or a higher capacity of cancer cells to mend DNA damage. Furthermore, cisplatin is associated with numerous side effects, with diarrhea being a common complication [5]. This side effect stems from reduced sodium and fluid absorption in the intestines [6] a process regulated by the Na + /K + ATPase, or Na + /K+ pump. Situated in the basolateral membrane of intestinal epithelial cells, this ATPase maintains the sodium gradient essential for electrolytes and secondary active transport processes. Notably, it has been identified as a contributor to cancer initiation, and a reduction in its activity has been recognized as a hallmark of apoptosis [7–9] and a causative agent of the cisplatin induced nephrotoxicity [10–12]. Since the ATPase plays overlapping roles in the kidney and colon, maintaining ion gradients, epithelial polarity, and barrier integrity, the observed cisplatin-induced disruption in its activity in renal tissue may plausibly extend to colonic epithelium, affecting electrolyte and water absorption and epithelial homeostasis. In fact, whether cisplatin modulates the activity of colonic Na ⁺ /K ⁺ -ATPase remains till now an open question, and data on this topic are still scarce. This gap in knowledge forms the foundation of the present research, which employs Caco-2 cells as a model to investigate whether cisplatin directly or indirectly targets colonic Na^+^/K^+^ ATPase. Additionally, this study aims to delineate the underlying mechanisms involved, providing insights that may alleviate some of the drug’s adverse effects by targeting relevant mediators.

## Materials and methods

### Materials

Cisplatin and the p38 MAPK inhibitor, doramapimod, were purchased from Abcam, Cambridge, UK. The primary mouse monoclonal antibody for GAPDH (cat #sc-47724 used at a dilution of 1:150) and the Goat anti-mouse horseradish peroxidase (HRP) conjugated secondary IgG (cat # sc-516102 used at a dilution of 1:4000) were purchased from Santa Cruz Biotechnology, CA, USA. Biorad protein reagent, nitrocellulose membranes, and western blotting luminol reagent were obtained from Biorad, California, USA. The JNK inhibitor (SP600125) and RpcAMP were purchased from Tocris Bioscience (Abingdon,Oxford, UK).The ERK inhibitor, PD98059, and the calcium chelator, BAPTA-AM (CAS-126150-97-8), were procured from Calbiochem, CA, USA.

Ouabain, Dulbecco’s Minimal Essential Medium (DMEM) with 4500 mg/L glucose and pyridoxine HCL, trypsin-EDTA, Penicillin/Streptomycin, Fetal Bovine Serum (FBS), 10x Phosphate Buffered Saline (PBS) without magnesium and calcium, Adenosine 5′-triphosphate disodium salt (ATP), 2’-O-Dibutyryladenosine 3′,5′-cyclic monophosphate sodium salt (dbcAMP), a purified Adenosine Triphosphatase (ATPase) from porcine cerebral cortex and the mouse monoclonal anti-Na^+^/K^+^ ATPase α1 (cat # ZMS1029, used at 1:5000 dilution) were procured all from Sigma, Chemical Co, St Louis Missouri, USA.

The Caco-2 cell line (male human colonic adenocarcinoma cell line, RRID: CVCL_0025**)** was obtained in 2021 from the American Type Culture Collection (ATCC). Cells were authenticated by ATCC and certified to be mycoplasma-free upon receipt. Later on, routine mycoplasma screening was conducted every 6 weeks in our lab.

All other chemicals were purchased from Sigma, Chemical Co, St Louis Missouri, USA.

### Methods

#### Culture and collection of Caco-2 cells.

Caco-2 cells, at passages 32–50, were cultured in DMEM supplemented with 1% penicillin, 1% streptomycin, and 10% fetal bovine serum (FBS). Cells were seeded in six-well plates at a density of 150,000 cells per well and maintained in a humidified incubator (95% O₂, 5% CO₂) at 37°C. Treatments were performed when cells reached 85–90% confluence following overnight starvation. After treatment, cells were washed with PBS buffer (pH 7.4) and collected using lysis buffer containing protease inhibitors. The lysates were homogenized and centrifuged at 20,000g for 30 minutes at 4°C. Proteins in the supernatant were quantified using the Bradford assay. These protein samples were then utilized for assessing Na^+^/K^+^ ATPase activity and conducting western blot analysis.

#### Cell viability test.

At 60% confluence, Caco-2 cells were starved overnight and treated the next day with 8µM cisplatin for 24 hrs. The cells were washed with PBS, trypsinized, suspended in 1 ml DMEM, and spun at 206g and 4°C for 5 minutes. The pelleted cells were then re-suspended in 1 ml DMEM. Equal volumes of cell suspension and trypan blue were mixed and aliquots were used to count the cells (viable/dead) using a hemocytometer.

#### Na^+^/K^+^ ATPase activity assay.

The activity of the Na^+^/K^+^ ATPase was assayed as described by Esmann [13] by measuring the amount of inorganic phosphate liberated in the presence and absence of ouabain, a specific inhibitor of the ATPase. Following protein quantification in the supernatant obtained by centrifugation of the cell lysate (as previously described), protein concentrations were adjusted to 0.5 μg/μl using histidine buffer (pH 7.4, 150 mM). Samples containing phosphatase inhibitors (2.7 mM pyrophosphate and 2.7 mM glycerophosphate) were incubated with 0.2% saponin at room temperature for 30 minutes. Subsequently, aliquots were taken and incubated for 10 minutes at 37 °C in histidine buffer supplemented with NaCl (121.5 mM), KCl (19.6 mM), and MgCl₂ (3.92 mM), both in the presence and absence of ouabain (1.47 mM). Adenosine triphosphate (ATP; 10 mM) was then added (final concentration 2.94 mM), and the samples were incubated for an additional 30 minutes. The reaction was terminated by adding an equal volume of 50% trichloroacetic acid, followed by centrifugation at 3000 g for 5 minutes. Inorganic phosphate released into the supernatant was quantified colorimetrically at 750 nm, according to the method described by Taussky & Shorr [14]. The results are reported as a percentage of the control values.

#### Preparation of cells with GFP-tagged Na^+/^K^+^ ATPase.

Caco-2 cells were stably transfected with GFP-tagged Na^+^/K^+^ ATPase α-1 using a plasmid generously provided by the Sznajder Laboratory at Northwestern University and the membrane marker mCherry-Mem (Addgene plasmid #55779), kindly gifted by Catherine Berlot [15]. The wheat germ agglutinin Alexa Fluor ^TM^ 594 was acquired from Invitrogen, OR, USA.

#### Treatment of Caco2 cells.

In all treatments with cisplatin, an equal volume of the vehicle, 0.9% NaCl, was added to the control.

*Dose and time-response study on the effect of cisplatin on the Na*^*+*^*/K*^*+*^ *ATPase activity*: Following an overnight starvation period, Caco-2 cells were treated with various concentrations of cisplatin (2, 4, 6, 8, and 10 µM) for 12 or 24 hours and the activity of Na^+^/K^+^ ATPase was subsequently measured. Changes in activity at 24 hours mirrored those observed at 12 hours. Notably, the highest enzymatic change was observed at 8 µM. Consequently, this concentration and a treatment period of 24 hours were adopted for all subsequent experiments.

*The influence of the Na-K-2Cl cotransporter (NKCC) and the coupled Na*^*+*^*/H*^*+*^ *and Cl*^*-*^*/HCO3*^*-*^ *exchangers on the effect of cisplatin on the Na*^*+*^*/K*^*+*^ *ATPase:* Cisplatin-resistant cells were found to feature higher intracellular chloride concentrations. To test whether changes in intracellular chloride levels could affect the cisplatin-induced changes in the ATPase activity, Caco-2 cells were treated with 600µM furosemide, an inhibitor of the NKCC cotransporter, or with 100µM amiloride, an inhibitor of the exchangers Na^+^/H^+^.

*Investigating a possible direct effect of cisplatin on the sodium-potassium ATPase:* The effect of cisplatin (8µM) on the activity of a purified porcine cerebral cortex Na^+^/K^+^ ATPase (50 µg) was assayed as described before by directly incubating the enzyme in the presence of the drug for 20 minutes at 37°C in a histidine buffer containing all the substrates described before.

*The effect of cisplatin on the membrane abundance of the Na*^*+*^*/K*^*+*^ *ATPase*: Caco-2 cells expressing GFP-tagged Na^+^/K^+^ ATPase (50,000 cells) were seeded onto Poly-D-Lysine-coated glass-bottomed dishes and incubated for 24 hours in DMEM supplemented with FBS, penicillin, and streptomycin. After an overnight starvation period, the cells were treated with 8 µM cisplatin for 24 hours. Following treatment, the cells were washed with PBS buffer (pH 7.4), fixed with 500 µL of 4% paraformaldehyde, and stained with Alexa Fluor™ Wheat Germ Agglutinin (WGA) dye to label the plasma membrane.

Imaging was performed using an inverted immunofluorescence microscope equipped with a × 40 objective lens. Specific excitation wavelengths for GFP and WGA were used (FS 14 and FS 10, respectively). The images were captured and scaled using AxioVision software, and subsequent image processing was carried out with ImageJ software. Cells displaying GFP and WGA expression were analyzed, and the green-to-red fluorescence ratio was calculated.

#### Western blot analysis.

Fifty micrograms of cell homogenate proteins were run on 10% SDS polyacrylamide gels transferred to nitrocellulose membranes and then cut into two strips containing each the Na^+^/K^+^ ATPase or the GAPDH proteins. They were then incubated overnight at 4°C with a primary anti-Na^+^/K^+^ ATPase α1 antibody, or an anti-GAPDH followed by an incubation with a goat anti-mouse HRP conjugated IgG secondary antibody for 1 hour at room temperature. The signal was detected using the ChemiDoc™ MP Imaging System with the Clarity™ ECL Substrate (luminol-based).

#### Determination of the components of the signaling cascade.

*Involvement of JNK, ERK, p38 MAPK, PKA and calcium in the effect of cisplatin on the Na*^*+*^*/K*^*+*^ *ATPase activity:* Caco-2 cells were treated for 24 hrs and after an overnight starvation with 8µM cisplatin in presence and absence of inhibitors of JNK (SP600125,50µM), ERK (PD98059, 50µM), p38 MAPK (doramapimod, 40nM), or PKA (RpcAMP, 30µM). All inhibitors were added 30 min before cisplatin.

The involvement of JNK was further investigated by treating the cells with cisplatin in the presence of anisomycin (250 ng/ml), a JNK activator. Anisomycin was added 30 minutes prior to cisplatin treatment.

To investigate any possible role of calcium in this pathway, the cells were pre-treated, 30 minutes before cisplatin, with BAPTA-AM (10µM) a calcium chelator, or with ionomycin (6µM), a calcium ionophore.

*Locating the involved molecules with respect to each other*: The position of p38 MAPK with respect to PKA was determined by treating the cells with 8µM cisplatin after a 30 min simultaneous pre-treatment with an inhibitor of p38 MAPK (doramapimod, 40nM), and an activator of PKA (dibutyryl cyclic adenosine mono phosphate, dbcAMP, 10µM). The Caco-2 cells were then incubated for 24 hours, and processed for the ATPase activity assay.

The relationship between PKA with JNK was determined by studying the effect of cisplatin (8µM, 24 hrs) when PKA and JNK were inhibited simultaneously with respectively RpcAMP (30µM) and SP600125 (50µM). The inhibitors were added 30 min before cisplatin.

To determine if calcium is upstream or downstream PKA the activity of the ATPase was examined in cells treated 30 min before cisplatin with a calcium chelator BAPTA-AM (10µM), and a PKA activator dbcAMP (10µM). Cells were also treated with cisplatin in the simultaneous presence of the calcium ionophore ionomycin (6µM) and the p38 MAPK inhibitor doramapinod (40nM)

#### Statistical analysis.

The data were tested for statistical significance using a t- test or a one-way analysis of variance followed by a Tukey-Kramer multiple comparison test using GraphPad InStat3. The results are reported as mean ± SEM.

## Results

### Dose- and time-dependent inhibition of Na ⁺ /K ⁺ -ATPase by Cisplatin

**Fig 1:**
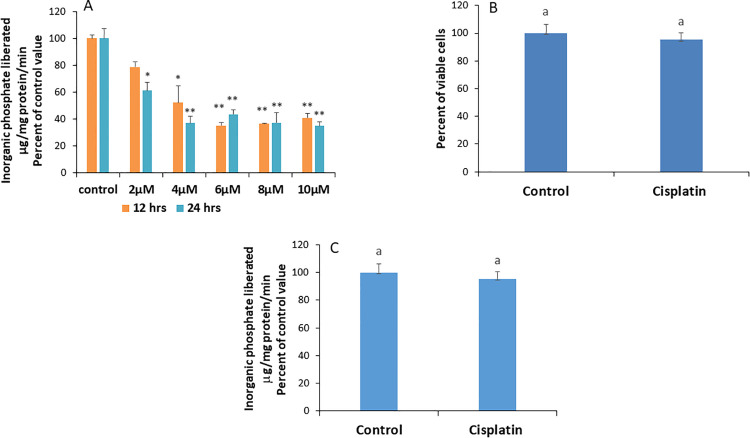
A) Dose- and time-dependent effects of cisplatin on Na^+^/K^+^ pump activity in cells treated for 12 or 24 hours. Data are presented as means ± SEM from 3 observations. Statistical significance compared to the control ***P < 0.001, *p < 0.01, determined by ANOVA followed by Tukey’s test. B) Cell viability following treatment with 8 µM cisplatin for 24 hours. Data are shown as means ± SEM from 3 observations. Bars with the same letter on top are not significantly different from each other, as determined by an unpaired t-test. p < 0.001. C) Effect of cisplatin on purified Na^+^/K^+^ ATPase. Data are presented as means ± SEM from 3 observations. Bars with the same letter on top are not significantly different from each other, as determined by an unpaired t-test. p < 0.01.

Treating Caco-2 cells for 12 or 24 hours with various sub-toxic concentrations of cisplatin revealed a dose-dependent inhibition of the Na^+^/K^+^ ATPase that appeared at 4 µM and was maintained at 6, 8, and 10µM across both treatment periods, although the highest inhibitory effect was observed at 8µM and 24 hrs with a residual activity of around 37% (Fig 1A). Consequently, this concentration and time period were adopted in all subsequent experiments.

### Cisplatin does not affect cell viability

To confirm that the cells remained alive throughout the 24-hour treatment period and to rule out the possibility that reduced activity was due to cell death, a viability test was performed. The results revealed comparable cell viability in the presence and absence of 8 µM cisplatin after 24 hours (Fig 1B).

### Cisplatin does not act directly on the Na^+^/K^+^ ATPase

To determine whether cisplatin directly interacts with the enzyme, its effect on purified Na ⁺ /K ⁺ -ATPase was evaluated. No change in activity was detected, suggesting that cisplatin does not inhibit the enzyme via direct binding (Fig 1C).

### Cisplatin induces a decrease in the abundance of Na^+^/K^+^ ATPase units at the plasma membrane

**Fig 2:**
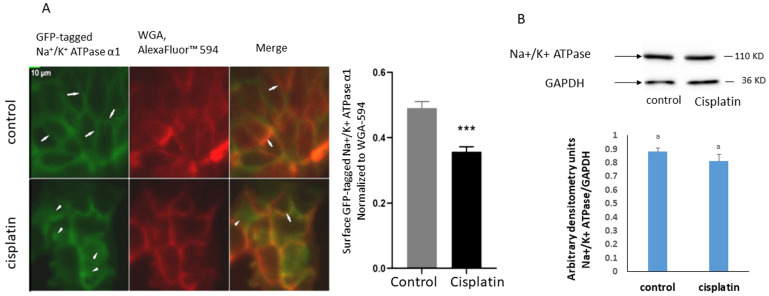
A) Fluorescence images of Caco-2 cells co-expressing GFP-tagged Na + /K + α1 subunits and mCherry-membrane marker after treatment with cisplatin for 24 hours. Statistical differences from the control were tested using a t-test (p < 0.01). B) Protein expression of the Na^+^/K^+^ ATPase in homogenates of cells treated with 8 µM cisplatin for 24 hours. The blots are representative of an experiment repeated 4 times. Values are normalized to GAPDH. Bars not sharing a common letter are considered significantly different from each other at p < 0.01 as indicated by a t- test.

To assess whether the reduction in the activity of the Na^+^/K^+^ ATPase is due to a decrease in its membrane abundance, fluorescence imaging was performed in Caco-2 cells expressing GFP-tagged Na ⁺ /K ⁺ -ATPase α1. The plasma membrane was labeled using wheat germ agglutinin Alexa Fluor ^TM^ 594. The relative quantification of GFP/ Alexa Fluor ^TM^ 594 showed a significant cisplatin-induced decrease in the ATPase residence at the membrane (Fig 2A).

### Na^+^/K^+^ ATPase molecules are internalized but not degraded

The reduced membrane abundance of Na⁺/K⁺-ATPase may result from either decreased protein expression or increased degradation. To investigate these possibilities, western blot analysis of whole-cell homogenates was performed. No significant change in total Na⁺/K⁺-ATPase levels was observed, indicating that the pumps were internalized and not degraded (Fig 2B).

### The Na-K-2Cl cotransporter and Na^+^/H^+^ exchanger modulate Cisplatin’s effect on Na^+^/K^+^ ATPase

**Fig 3:**
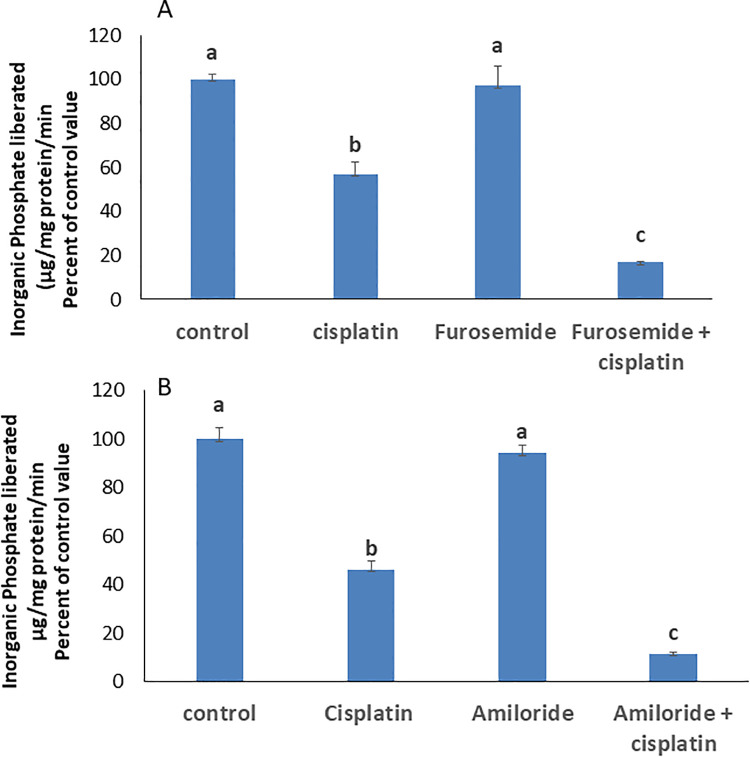
Effect of A) furosemide (600µM), and B) amiloride (100 µM) on the activity of the Na^+^/K^+^ ATPase in cells treated with 8µM cisplatin for 24 hrs. Data are presented as means ± SEM from 3 observations. Bars sharing the same letter are not significantly different as determined by ANOVA followed by Tukey’s test. p < 0.01.

To explore the involvement of the Na-K-2Cl cotransporter (NKCC) and Na + /H+ exchanger in the cisplatin’s effect, cells were co-treated with furosemide (NKCC inhibitor) or amiloride (Na ⁺ /H⁺ exchanger inhibitor). Both treatments further reduced Na ⁺ /K ⁺ -ATPase activity by respectively 40% and 35% (Fig 3A & B).

### Mediators of cisplatin effect on the Na^+^/K^+^ ATPase

Cisplatin interacts with multiple signaling pathways involving mediators such as calcium and various kinases like the Mitogen-Activated Protein Kinases (MAPKs), p38 MAPK, c-Jun N-terminal Kinase (JNK), as well as Protein Kinase A (PKA). These interactions significantly influence cisplatin’s effects on both cancerous and normal tissues. As a result, the specific roles and impacts of each of these signaling molecules were investigated.

**Fig 4:**
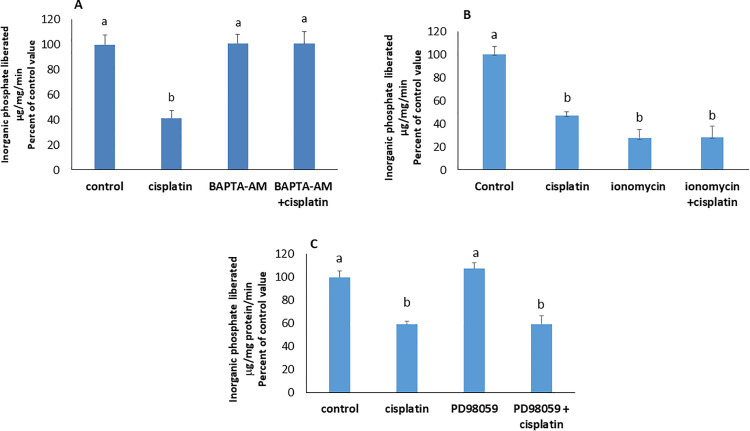
Effect of cisplatin in presence of A) the calcium chelator BAPTA-AM (10µM) and B) the calcium ionophore ionomycin (6µM) on the activity of the Na + /K + ATPase. Values are means ± SEM of 3 observations. Bars not sharing the same letter are considered significantly different from each other at p < 0.001, as indicated by ANOVA followed by Tukey test.

*Calcium Signaling Mediates Cisplatin-Induced Inhibition*: Calcium chelation with BAPTA-AM (10µM) abolished cisplatin’s effect, indicating its involvement in the induced inhibition of the ATPase (Fig 4A). Treating the cells with the calcium ionophore ionomycin (6µM) reduced the activity of the Na^+^/K^+^ ATPase down to around 27% of its control value. No further reduction was observed in the simultaneous presence of cisplatin (Fig 4B) suggesting that calcium is along the pathway activated by cisplatin.

C. effect of cisplatin on the ATPase in presence of PD98059 an ERK inhibitor. Values are means ± SEM of 3 observations. Bars not sharing a common letter are considered significantly different from each other at p < 0.01, as indicated by ANOVA followed by Tukey test.

*ERK signaling is not involved in cisplatin’s effect*: To evaluate ERK’s role, the activity of the pump was measured in Caco-2 cells treated with cisplatin in presence of PD98059 (a MEK/ERK inhibitor). The ATPase activity was not significantly different from that of the control. ERK’s inhibition did not negate the inhibitory effect of cisplatin (Fig 4C), inferring that cisplatin acts independently of ERK.

**Fig 5:**
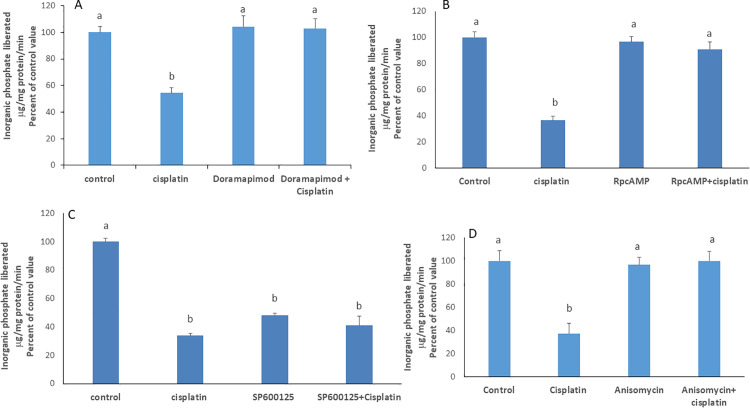
Effect of cisplatin in presence of A) doramapimod (40nM), a p38 MAPK inhibitor; B) RpcAMP (30µM), a PKA inhibitor; C) SP600125, a JNK inhibitor; D) anisomycin, a JNK activator; Values are means ± SEM of 3 observations. Bars not sharing the same letter are considered significantly different from each other at p < 0.01, as indicated by ANOVA followed by Tukey test.

*p38 MAPK and PKA mediate cisplatin’s inhibitory effect*: In presence of doramapimod (40nM), or RpcAMP (30µM), respective inhibitors of p38 MAPK and PKA, cisplatin did not induce its inhibitory effect on the ATPase (Figs 5 A & B) indicating their participation in the signaling cascade.

*Cisplatin inhibits the Na*^*+*^*/K*^*+*^ *ATPase by inhibiting JNK*: The inhibition of JNK alone with SP600125(50 µM) induced, like cisplatin, a decrease in the activity of the Na^+^/K^+^ ATPase that was maintained in the simultaneous presence of the drug (Fig 5C). The effect of cisplatin was not manifested in presence of anisomycin, a JNK activator (Fig 5D). The results suggest that cisplatin inhibits the Na^+^/K^+^ ATPase by suppressing JNK activity.

**Fig 6:**
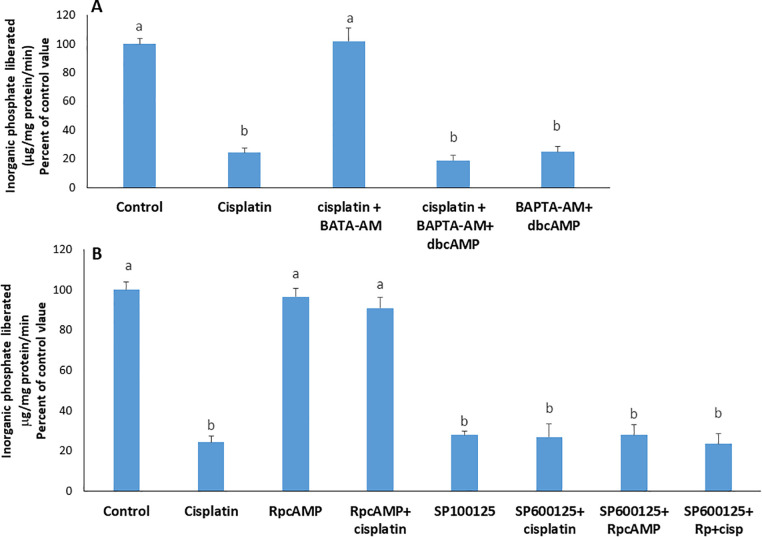
Effect of cisplatin on the Na^+^/K^+^ ATPase in A) the simultaneous presence of BAPTA-AM(10 µM) and dbcAMP (10 µM); B) the simultaneous presence of an inhibitor of JNK (SP600125) and an inhibitor of PKA (Rp cAMP). Values are means ± SEM of 3 observations. Bars not sharing the same letter are considered significantly different from each other at p < 0.05, as indicated by ANOVA followed by Tukey test.

*Signaling hierarchy: calcium, PKA, and JNK*: To define the signaling hierarchy, cells were co-treated with BAPTA-AM and dbcAMP. While cisplatin could not exert any effect on the ATPase in presence of the calcium chelator BAPTA-AM (10 µM), the simultaneous addition of dbcAMP (10µM), a PKA activator, restored the inhibitory effect of cisplatin, implying that PKA is downstream of calcium (Fig 6A).

Inhibition of PKA with RpcAMP blocked the effect of cisplatin. This effect reappeared upon the simultaneous addition of SP600125, an inhibitor of JNK, indicating that PKA is upstream JNK, and inhibits it (Fig 6B).

## Discussion

Cisplatin induces cell damage and triggers apoptosis [3]. Since apoptosis is known to reduce the activity of the Na^+^/K^+^ ATPase [16], we opted to study the effect of sub-toxic levels of the drug on the activity of the enzyme and to determine, if such an effect exists, the mediators involved. At 6–10 µM, cisplatin significantly inhibited pump activity without compromising cell viability, supporting the reported link between cisplatin’s apoptotic effects and ATPase dysfunction. These results suggest that gastrointestinal side-effects of cisplatin may stem in part from colonic ATPase dysfunction, opening avenues for gut-protective interventions. These results may offer insight into some of the drug’s undesirable side effects, such as diarrhea and constipation, which are associated with dysregulation of the pump [5].

Cisplatin uptake remains debated, with evidence for both passive diffusion and copper transporter-mediated entry [3]. Its short-lived efficacy is often attributed to resistance mechanisms, including reduced cytoplasmic accumulation, which depends on intracellular chloride levels [17]. Salerno *et al.* [17] reported that cisplatin uptake is enhanced under conditions of reduced intracellular Cl ⁻ . Intracellular Cl⁻ levels are maintained primarily by NKCC1 together with the coupled Cl ⁻ /HCO₃⁻ and Na ⁺ /H⁺ exchangers. Pharmacological inhibition of these transporters using furosemide and amiloride, which lowers intracellular Cl⁻ and increases the Cl transmembrane gradient, potentiated further cisplatin’s inhibitory effect on the Na ⁺ /K ⁺ -ATPase. This observation is consistent with increased cellular cisplatin uptake driven by the Cl⁻ gradient, thereby attributing the greater ATPase inhibition to enhanced drug accumulation.

To determine whether cisplatin acts directly on the ATPase, we tested its effect on the purified enzyme. No change in activity was observed, indicating an indirect mechanism. Although the literature reports an interaction of cisplatin with five cysteine residues (C452, C456, C457, C577, and C656) within the intracellular loop connecting transmembrane helices 4 and 5 [10,11], these findings were based on studies using the isolated C45 loop, rather than the full enzyme which may influence the accessibility and reactivity of these residues, limiting the physiological relevance of these observations.

The observed inhibition of the Na + /K + ATPase could result from reduced membrane abundance, a decreased specific activity, or both. Imaging of GFP-tagged ATPase revealed fewer membrane-localized units while western blot analysis showed unchanged total protein levels. The results infer that the ATPase units were internalized but did not undergo degradation.

Cisplatin’s apoptotic effects involve kinases such as ERK, JNK, p38 MAPK [18–21], and PKA [22], as well as calcium signaling [23]. We examined their roles in ATPase regulation.

ERK inhibition with PD98059 did not alter cisplatin’s effect, suggesting that ERK is not involved. Notably, ERK’s role in apoptosis is context-dependent, promoting survival in some cell lines (e.g., SK-OV-3 [24]) and apoptosis in others (e.g., osteosarcoma, neuroblastoma [25,26]).

In contrast, inhibition of p38 MAPK and PKA blocked cisplatin’s effect, confirming their involvement [22,27–29]. However, JNK inhibition, even in the absence of cisplatin, significantly reduced the pump’s activity, suggesting that JNK is constitutively active and plays a basal role in maintaining the basal activity of the Na + /K + ATPase. This finding was further supported by the stimulation observed in cells treated with the JNK activator, anisomycin. Furthermore, JNK inhibition in the presence of cisplatin did not lead to a greater inhibition, implying that JNK operates along the pathway activated by cisplatin. A greater inhibition would have been expected, had JNK been acting independently of this pathway.

The relationship between JNK and cisplatin-induced apoptosis is context-dependent. Recent studies describe JNK as a double-edged sword, playing a crucial role in promoting cell death while also contributing to enhanced resistance to cisplatin by activating cell survival mechanisms [30].

The effect of cisplatin was abolished by p38 MAPK inhibition, yet restored upon co‑administration of dbcAMP, indicating that PKA functions downstream of p38 MAPK. Consistently, inhibition of PKA with RpcAMP abrogated the cisplatin response, but this effect was reversed when JNK was simultaneously inhibited, supporting a model in which JNK operates downstream of PKA. Together, these observations delineate a signaling cascade in which cisplatin engages p38 MAPK, leading to PKA activation and subsequent modulation of JNK activity.

Cisplatin failed to exert its effect under calcium chelation, highlighting calcium’s role in Na ⁺ /K ⁺ -ATPase inhibition and the activation of PKA and p38 MAPK. Consistently, treatment with BAPTA-AM abolished the drug’s activity, which reappeared upon co-administration of dbcAMP. Likewise, the inhibitory action of the calcium ionophore ionomycin was suppressed in the presence of the p38 MAPK inhibitor doramapimod.

These findings suggest that PKA is downstream of calcium and p38 MAPK, with all three mediators acting along the same signaling pathway.

Calcium regulates apoptosis by modulating intracellular levels through channels, pumps, exchangers, and binding proteins [23,31–33]. Calcium-induced activation of p38 MAPK has been linked to CaMKII and ASK1/MAP3K5, which trigger apoptosis under stress [34–36].

It can be concluded that cisplatin inhibits Na ⁺ /K ⁺ -ATPase via a signaling cascade involving regulators of apoptosis. PKA and p38 MAPK are activated, while JNK is inhibited. JNK’s dual role in apoptosis and resistance is well-documented, and p38 MAPK knockout studies show increased JNK activity [37,38]. Our findings suggest that PKA mediates JNK inhibition, consistent with reports of cAMP reducing JNK phosphorylation via CREB-induced c-FLIP(L) and MKP-1 [39,40]. JNK’s stimulatory effect on Na ⁺ /K ⁺ -ATPase has also been observed in LLC-PK1 cells [41].

**Fig 7:**
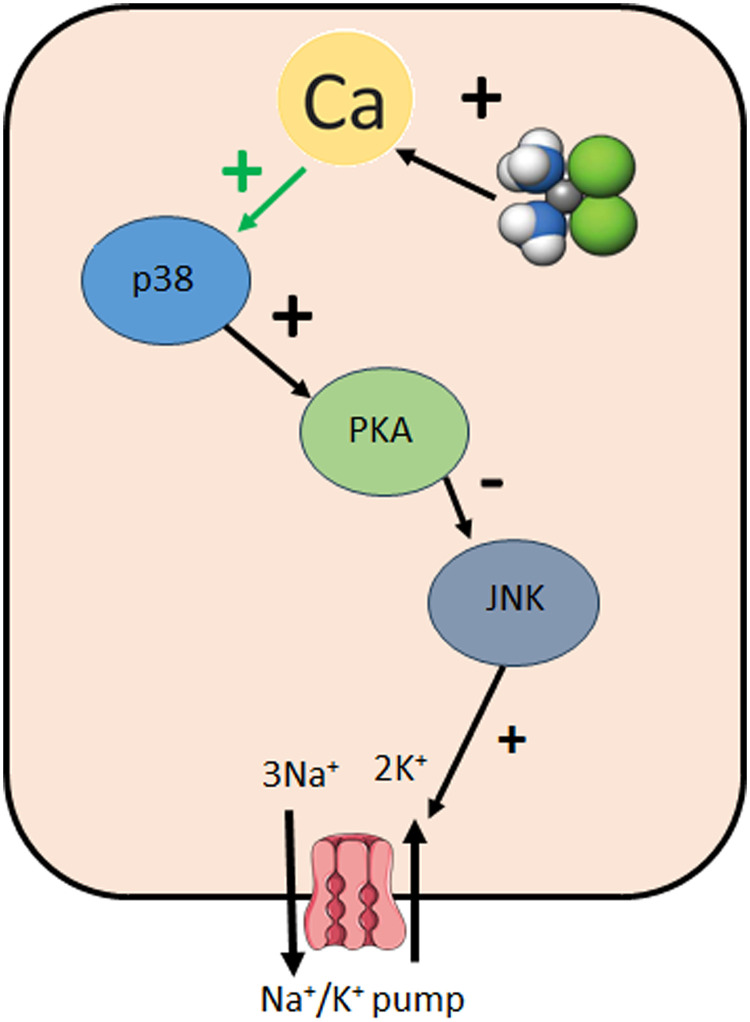
The signaling pathway mediating the effect of cisplatin on the Na + /K + ATPase.

We propose that cisplatin uptake is driven by the chloride gradient. Once inside the cell, it elevates intracellular calcium, activates p38 MAPK and PKA, and inhibits JNK, leading to reduced Na ⁺ /K ⁺ -ATPase activity. This pathway is summarized in Fig 7.

## Supporting information

S1 Fig Uncropped raw western blot images showing protein expression of Na + /K + ATPase and GAPDH. (PDF)

S2 File Raw data for all experiments. (XLSX)
