# Supplementary material for: Mechanism of action of cisplatin on Na+/K+ ATPase of Caco-2 colon cells in vitro
Source: PLoS One. 2026 Feb 24;21(2):e0342707. doi: 10.1371/journal.pone.0342707 (PMC12931807; doi:10.1371/journal.pone.0342707)

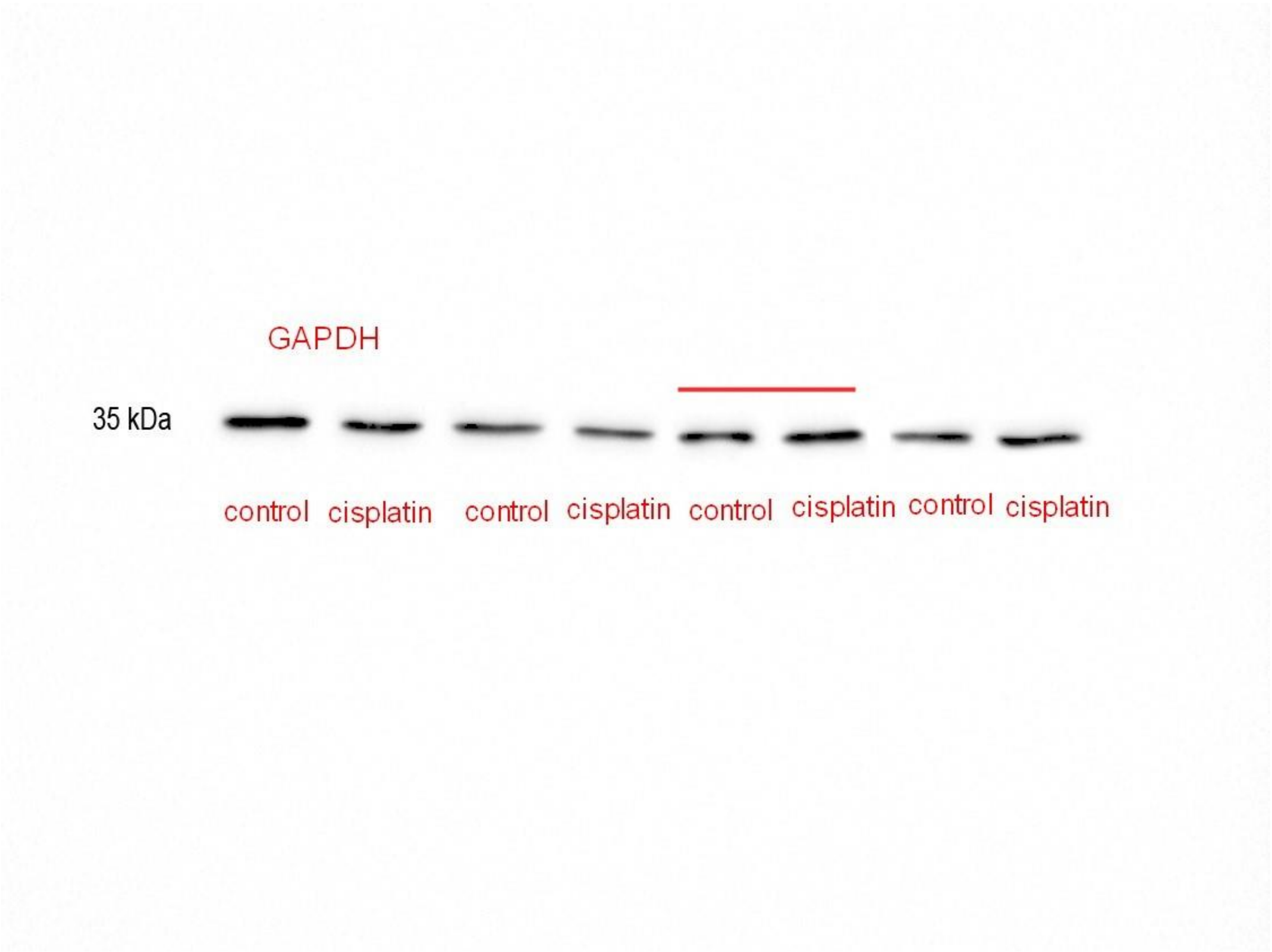

Figure 2B : original uncropped images of GAPDH bands

Figure 2B: original uncropped images of Na+/K+ ATPase bands

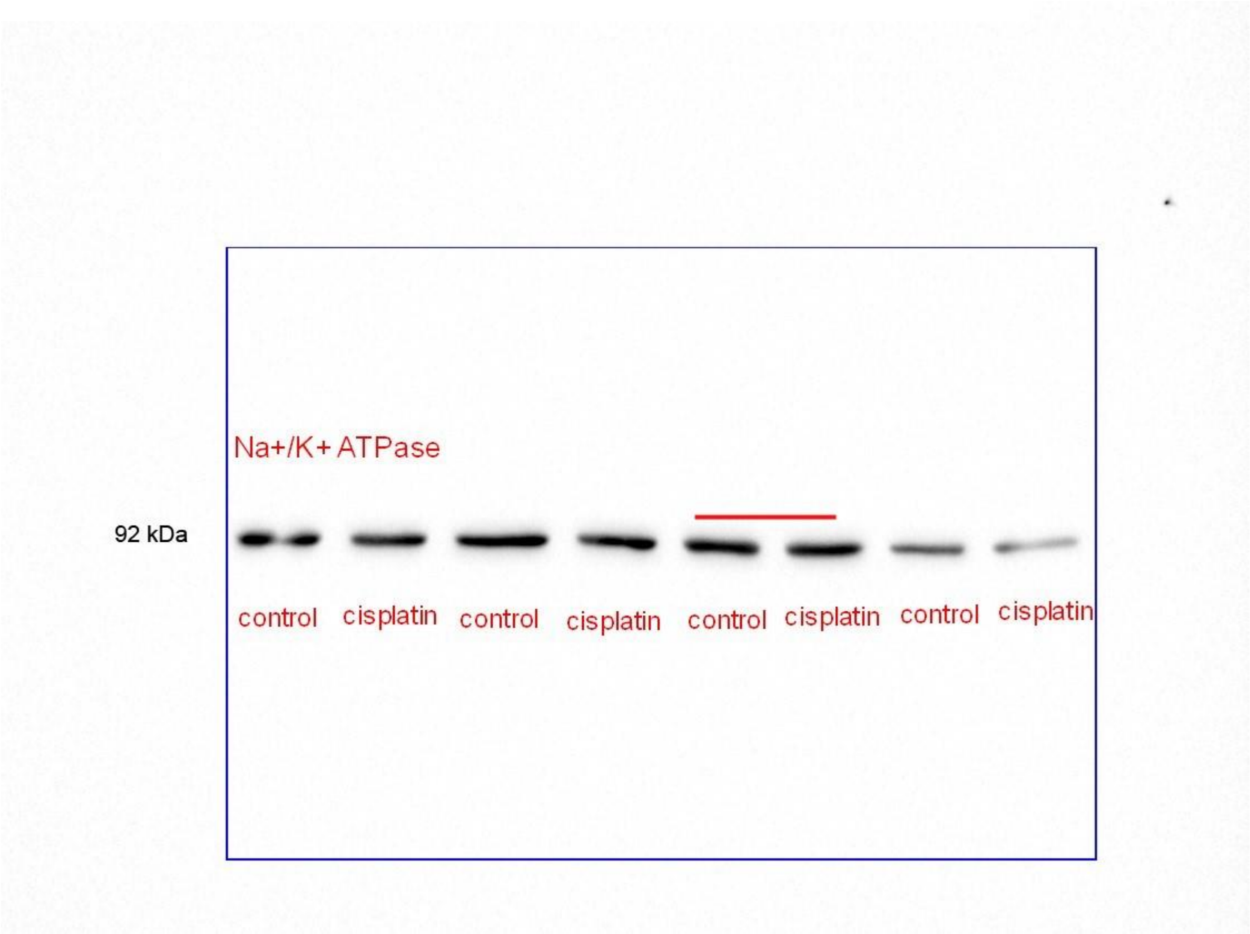

Supplement: S1 Fig — (PDF) [file pone.0342707.s001.pdf]
